# Supplementary material for: How do paediatric physical therapists teach motor skills to children with Developmental Coordination Disorder? An interview study
Source: PLoS One. 2024 Feb 1;19(2):e0297119. doi: 10.1371/journal.pone.0297119 (PMC10833570; doi:10.1371/journal.pone.0297119)
Supplement: S1 File — (DOCX) [file pone.0297119.s001.docx]

# **S1 File.**

# **Motor learning strategies commonly described in literature**

| **Types of motor learning** | |
| --- | --- |
| Implicit and explicit motor learning | Implicit motor learning: learning which progresses with no or minimal increase in verbal knowledge of movement performance (e.g. facts and rules) and without awareness. Implicitly learned skills are (unconsciously) retrieved from implicit memory [1]. |
|  | Explicit motor learning: learning which generates verbal knowledge of movement performance (e.g. facts and rules), involves cognitive stages within the learning process and is dependent on the working memory [1]. |
| **Instructions and feedback** | |
| Self-controlled | In self-controlled instructions and feedback the learner determines the timing or modality of the instructions and feedback, which enhances the learner’s autonomy [2]. |
| Enhanced Expectancies | Instructions and feedback that enhance the learner’s self-efficacy expectations (or confidence) about the movement performance [2]. |
| External and internal focus of attention | External focus: an external focus of attention directs the learner’s attention to the impact of the movement on the environment [3]. |
|  | Internal focus: an internal focus of attention directs the attention to the learner’s body movements [3]. |
| Knowledge of performance | Feedback providing the learner with information about its own body movements [4]. |
| Knowledge of results | Feedback after the performance providing the learner with information about its success in meeting the environmental goal [4]. |
| Observation learning | Watching a model performing a motor task, which provides the learner with a cognitive model of the movement performance [5]. |
| Analogy learning | Providing the learner with an analogy (metaphor) that integrates the complex structure of the to-be-learned task [6]. |
| **Organization of practice** | |
| Random and blocked practice | Random practice: practicing various motor tasks in a random order [7]. |
|  | Blocked practice: practicing the same motor tasks in a blocked order, without alternation with other motor tasks [7]. |
| Variable and constant practice | Variable practice: practicing a motor task with increased variation during practice [8]. |
|  | Constant practice: practicing a motor task repetitively without variation during practice [8]. |
| Whole and part practice | Whole practice: practicing a motor task in its entirety [9]. |
|  | Part practice: practicing units of motor tasks, after breaking down a motor task into smaller units [9]. |
| Dual-task learning | Using a secondary (mostly cognitive) task to draw the attention of the learner to, whereby short-memory capacity cannot be used for explicit knowledge of the primary task to-be-learned [10]. |
| Errorless learning | Arranging the practice situation in such way that the learner makes no or few outcome errors [11]. |
| Guided discovery | Guiding the learner to the correct movement response with a sequence of questions [12]. |
| Motor imagery | Asking the learner to mentally execute the motor task without actually doing it [13]. |
| Self-controlled | In self-controlled practice conditions the learner is provided with choice, which enhances the learner’s autonomy [2]. |
| Trial-and-error learning | The learner performs the task repeatedly and optimizes its performance with intrinsic and extrinsic feedback on its errors [14]. |

# **References**

1. Kleynen M, Braun SM, Bleijlevens MH, Lexis M a., Rasquin SM, Halfens J, et al. Using a Delphi technique to seek consensus regarding definitions, descriptions and classification of terms related to implicit and explicit forms of motor learning. PLoS One. 2014;9(6):1–11. doi: 10.1371/journal.pone.0100227
2. Wulf G, Lewthwaite R. Optimizing performance through intrinsic motivation and attention for learning: the OPTIMAL theory of motor learning. Psychon Bull Rev. 2016 Oct;23(5):1382–1414. doi: 10.3758/s13423-015-0999-9

3. Wulf G, Hoss M, Prinz W. Instructions for motor learning: differential effects of internal versus external focus of attention. J Mot Behav. 1998 Jun;30(2):169–79. doi: 10.1371/journal.pone.0100227

4. Salmoni AW, Schmidt RA, Walter CB. Knowledge of results and motor learning: a review and critical reappraisal. Psychol Bull. 1984;95(3):355–86. doi: 10.1371/journal.pone.0100227

5. Adams JA. Use of the model’s knowledge of results to increase the observer’s performance. Journal of Human Movement Studies. 1986;12:89–98.

6. Liao CM, Masters RSW. Analogy learning: a means to implicit motor learning. J Sports Sci. 2001;19(5):307–19. doi: 10.1371/journal.pone.0100227

7. Magill RA, Hall KG. A review of the contextual interference effect in motor skill acquisition. Hum Mov Sci. 1990;9(3–5):241–89. doi: 10.1371/journal.pone.0100227

8. Tassignon B, Verschueren J, Baeyens JP, Benjaminse A, Gokeler A, Serrien B, et al. An exploratory meta-analytic review on the empirical evidence of differential learning as an enhanced motor learning method. Front Psychol. 2021;12:533033. doi: 10.1371/journal.pone.0100227

9. Fontana FE, Furtado O, Mazzardo O, Gallagher JD. Whole and part practice: a meta-analysis. Percept Mot Skills. 2009 Oct;109(2):517–30. doi: 10.1371/journal.pone.0100227

10. Masters RSW. Knowledge, knerves and know‐how: the role of explicit versus implicit knowledge in the breakdown of a complex motor skill under pressure. British Journal of Psychology. 1992;83(3):343–58. doi: 10.1371/journal.pone.0100227

11. Maxwell JP, Masters RS, Kerr E, Weedon E. The implicit benefit of learning without errors. The Quarterly journal of experimental psychology . 2001;54:1049–68. doi: 10.1080/713756014

12. Mosston M, Ashworth S. Teaching physical education: first online edition [Internet]. 1st ed. Copyright 2012 Spectrum of Teaching Styles. Spectrum Institute for teaching and learning; 2008. 358 p. Available from: http://www.spectrumofteachingstyles.org/

13. Jeannerod M. Neural simulation of action: a unifying mechanism for motor cognition. Neuroimage. 2001;14:S103–9. doi: 10.1080/713756014

14. Jueptner M, Frith CD, Brooks DJ, Frackowiak RSJ, Passingham RE. Anatomy of Motor Learning. II. Subcortical Structures and Learning by Trial and Error. J Neurophysiol. 1997;77:1325–37. doi: 10.1080/713756014
