# Supplementary material for: How do paediatric physical therapists teach motor skills to children with Developmental Coordination Disorder? An interview study
Source: PLoS One. 2024 Feb 1;19(2):e0297119. doi: 10.1371/journal.pone.0297119 (PMC10833570; doi:10.1371/journal.pone.0297119)
Supplement: S2 File — (DOCX) [file pone.0297119.s002.docx]

**S2 File. Interview guide for the individual interviews**

*(Translated to English, interviews were conducted in Dutch)*

| **Instructions for the interviewer** | | | |
| --- | --- | --- | --- |
| - Welcome the therapist, and introduce yourself. - Mention explicitly that the aim is to gain insights into therapists’ use of motor learning strategies (MLSs) to teach motor skills to children with Developmental Coordination Disorder (DCD), and that you are interested in therapists’ experiences without judging their answers or actions. - Mention that all information shared during the interview will be in confidence and that privacy will be respected. The therapist can interrupt or end the interview whenever he or she wants as described in the information letter and consent form. - Use the interview guide flexibly. Feel free to switch between topics if the conversation gives rise to it. - Ask open-ended follow-up questions to invite the therapist to elaborate on their answers. Suggestions are included in this interview guide. - Invite the therapist to use lots of examples, or to simulate situations, to support their answers. - Refer to the video-taped treatment session of the therapist to encourage the therapist to elaborate on answers or specific examples observed. - Use prompts and probes to encourage the therapist to elaborate on their answers. - Start the two recording devices. | | | |
| **Topic list** | | | |
| **Topic** | **Introduction** | **Main questions** | **Follow-up questions and/or topics** |
| **Topic 1**: Therapists’ experiences in treating children with DCD and teaching (a)typical developing children motor skills | These questions are to get acquainted, and to get insights into your experiences. | Which experiences do you have in *treating* children with DCD? | - The experiences with the various needs that children with DCD have - The experiences with different types of children with DCD, e.g. with and without comorbidity |
|  |  | Which experiences do you have in *teaching* children with or without DCD motor skills? | - The therapists’ opinion on the relevance of motor teaching in children (with DCD) - Non-therapeutic experiences in using MLSs, e.g. as sports trainer |
| **Topic 2**: Therapists’ use of MLSs to teach children with DCD motor skills | In treating children with DCD, we try to teach new motor skills or to optimize acquired motor skills to these children. I would like to get some insights into the various MLSs you use in your treatment sessions. | How do you shape your MLSs when teaching children with DCD motor skills? | - The use of instructions and feedback - The organization of practice - The use of specific learning strategies - When do you choose to use [mentioned MLS]? - Can you give an example of using [mentioned MLS] in clinical practice? - On the video-taped treatment session I saw you using/doing/telling/showing [the action of the therapist], can you share your thoughts about why you did this? |
| **Topic 3**: Therapists’ use of implicit and explicit motor learning approaches | In literature, motor learning approaches distinguishes implicit and explicit motor learning approaches. I would like to get more insights into your use of these approaches. | Are you familiar with the terms implicit and explicit motor learning? | - When “yes”: Can you give a description of both implicit and explicit motor learning? - When “no”, give a description: Do you recognize using implicit and explicit motor learning approaches as you heard the description? - Can you give an example on how you shaped implicit/explicit motor learning during a treatment session of a child with DCD? |
|  |  | Which advantages and/or disadvantages do you experience using implicit and explicit motor learning approaches in children with DCD? | - Why do you experience this as a (dis)advantage? - Do you prefer an implicit or explicit motor learning approach in children with DCD and can you explain why? |
| **Topic 4**: The adaptation of MLSs to suit child, task and environmental characteristics | For each child we use different MLSs. Even within a child, we use different MLSs within or between treatment sessions. I would like to get more insights into which variables guide your choices when teaching motor skills to children with DCD. | Which child, task and environmental characteristics do you map during your therapeutic examination of children with DCD? | - Question specific child, task and environmental characteristics - Why are you interested in [mentioned characteristic]? - Do you map the same characteristics for each child with DCD? Why yes/no? |
|  |  | How do these characteristics guide your choices in the use of MLSs? | - Why does [mentioned characteristic] guide your choices? - Can you give an example on how you acted differently in case of [mentioned characteristic]? - On the video-taped treatment session I saw you using/doing/telling/showing [the action of the therapist], can you share your thoughts about why you did this in this specific situation? |
|  |  | During treatment sessions it can occur that you start using other MLSs, for instance, because results are not as you expected. Which characteristics are leading in this adaptation? | - Can you explain how [mentioned characteristics] changed your use of MLS? - How did you acted initially and what did change? Why? - On the video-taped treatment session I saw you changing from [the action of the therapist] to [the action of the therapist], can you share your thoughts about why you did this? |
|  |  | You adapt the use of MLSs to each child individually. Can you explain why? | - Why is adapting MLSs to each individual child important for you? - Which characteristic is leading in guiding your choices? |
| **Closing question** before thanking the therapist |  | - Do you have something to add to everything already discussed, for instance, a specific example of your daily practice that you find really illustrative for you as a therapist? |  |
