# Supplementary material for: How do paediatric physical therapists teach motor skills to children with Developmental Coordination Disorder? An interview study
Source: PLoS One. 2024 Feb 1;19(2):e0297119. doi: 10.1371/journal.pone.0297119 (PMC10833570; doi:10.1371/journal.pone.0297119)
Supplement: S3 File — (DOCX) [file pone.0297119.s003.docx]

**S3 File. Interview guide for the focus groups**

*(Translated to English, interviews were conducted in Dutch)*

| **Instructions for the interviewer** | | | |
| --- | --- | --- | --- |
| - Welcome the therapists, and introduce yourself. - Mention explicitly that the aim is to gain insights into therapists’ use of motor learning strategies (MLSs) to teach motor skills to children with Developmental Coordination Disorder (DCD) without reaching consensus, and that you are interested in the therapists’ experiences without judging their answers or actions. - Ask the therapists to respect the opinions of each other without judging. - Mention that all information shared during the interview will be in confidence and that privacy will be respected. - Use the interview guide flexibly. Feel free to switch between topics if the conversation gives rise to this. - Ask open-ended follow-up questions to invite the therapists to elaborate on answers. Suggestions are included in this interview guide. - Invite the therapists to use lots of examples, or to simulate situations, to support the answers. - Use prompts and probes to encourage the therapists to elaborate on answers. - Start the recording devices. | | | |
| **Topic list focus group 1** | | | |
| **Topic** | **Introduction** | **Main questions** | **Follow-up questions** |
| **Introduction** | I would like to start with a question to get acquainted with each other. Please introduce yourself and answer the following question. | What materials do you favour using in treatment sessions? |  |
| **Topic 1**: Therapists’ use of MLSs in various tasks | During your treatments, you practise various tasks with children. I would like to get more insight into how you use your MLSs in these tasks. | Which tasks do you practise a lot with children with DCD? | - How do you use your MLSs when practising [the task mentioned]? - What task characteristic determines which MLSs you use? - Do you always practise tasks the same way? Why or why not? |
| **Topic 2**: The information content of instructions and feedback | In the individual interviews, some therapists mentioned using short instructions and feedback, while others used more extensive ones with more details to teach children with DCD skills. I would like to hear how you address this. | How much detail do you give in your instructions and feedback? | - Why do you provide your instructions and feedback in that way? - Is the amount of detail always the same in your instructions and feedback? Why or why not? - Can you give an example? |
| **Topic 3**: Environmental factors guiding therapists’ use of MLSs | In the individual interviews, therapists talked about how the environment of the child (e.g. parent, school) influences motor learning but they talked less about how this influences their use of MLSs. | How do environmental factors influence your use of MLSs? | - Which MLSs do you use when [mentioned environmental factor] is present? - Do you always use the same MLSs in case of [mentioned environmental factor]? Why or why not? |
| **Topic 4**: The trade-off between the child’s experiences of success and failure in the intervention | In the individual interviews, some therapists talked about the importance of success experiences, while others mentioned that errors are needed to learn. | How do you use errors during practice? | - Can you elaborate on your thoughts? - What are your thoughts on success experiences? - Is it the same for all children? |
| **Topic 5**: The use of variation in the intervention (e.g. random practice) | In the individual interviews, therapists talked about how they gradually increased steps between exercises, and how they decreased or increased complexity within tasks. However, they talked less about the use of variation between or within tasks. For instance, random/blocked practice, and constant/ variable practice. | How do you use variation during practice? | - Why do you use it in that way? - Can you give an example on how you used variation during practice? |
| **Topic 6**: Therapists’ adaptation of MLSs to the child’s learning stage | In the individual interviews, the therapist elaborated on how the learning stage of a child (e.g. cognitive stage) influenced their use of MLSs. | How do you adapt your use of MLSs to the learning stage of a child? | - Why do you adapt in this way? - Can you give an example? - Do you always use the same MLSs in learning stages? Why or why not? |
| **Closing question** before thanking the therapists |  | - Do you have something to add to everything already discussed, for instance, a specific example of your daily practice that you find really illustrative for you as a therapist? |  |
| **Topic list focus group 2** | | | |
| **Topic** | **Introduction** | **Main questions** | **Follow-up questions** |
| **Introduction** | I would like to start with a question to get acquainted with each other. Please introduce yourself and answer following question. | Children with DCD have specific needs they wanted to practise: which needs do you like most? |  |
| **Topic 1**: Therapists’ adaptation of MLSs to child characteristics | In previous interviews, therapists talked about how characteristics of the child guided their use of MLSs. I would like to explore that topic with you. | Which child characteristic do you take into consideration when determining what MLSs to use? | - How does [mentioned child characteristic] influence your use of MLSs? - Do you always practise the same when [mentioned child characteristic] is present? - Can you give an example? |
| **Topic 2**: Therapists’ use of MLSs in various tasks | In previous interviews, therapists talked about how they used MLSs in specific tasks. I would like to explore that topic with you. | Which task characteristic do you take into consideration when determining what MLSs to use? | - What make tasks more or less complex? - How does [mentioned task characteristic] influence your use of MLSs? - Can you give an example? |
| **Topic 3**: The interaction of child, task and environment | In previous interviews, therapists mentioned that the interaction of child, task and environment influenced their choice in MLSs. | What are your perspectives on that? | - Which characteristics within the child (or task, or environment) are most prominent in your choices? - Can you elaborate on your thoughts? |
| **Closing question** before thanking the participant |  | - Do you have something to add to everything already discussed, for instance, a specific example of your daily practice that you find really illustrative for you as a therapist? |  |
