# Supplementary material for: How do paediatric physical therapists teach motor skills to children with Developmental Coordination Disorder? An interview study
Source: PLoS One. 2024 Feb 1;19(2):e0297119. doi: 10.1371/journal.pone.0297119 (PMC10833570; doi:10.1371/journal.pone.0297119)
Supplement: Attachment — Submitted filename: Response to reviewers.pdf [file pone.0297119.s005.pdf]

Author’s response to decision letter for PONE-D-22-31852R1

How do therapists teach motor skills to children with Developmental Coordination Disorder? An interview study

Diepenbeek, 15-12-2023

Dear editor in chief, dear Prof. Dr. C. Capio,

Please find uploaded our second revision of the manuscript entitled: “How do therapists teach motor skills to children with Developmental Coordination Disorder? An interview study”

We revised our manuscript as requested in your email of November 13, 2023.

We would like to thank the editor and the reviewers for their feedback, and for giving the opportunity to revise our manuscript. Below we provide a point-to-point reply to each of the comments (in italic blue coloured). In the manuscript, we highlighted the changes by colouring the text blue, and the deleted text that was not replaced with track-changes.

We hope that with this revision and reply all concerns are satisfactorily addressed and the manuscript can be accepted for publication. Of course, we will be happy to answer any additional questions from the editorial office or reviewers.

Yours sincerely,

Ingrid van der Veer (first author, on behalf of all co-authors)

General comments:

Journal Requirements:

Please review your reference list to ensure that it is complete and correct. If you have cited papers that have been retracted, please include the rationale for doing so in the manuscript text, or remove these references and replace them with relevant current references. Any changes to the reference list should be mentioned in the rebuttal letter that accompanies your revised manuscript. If you need to cite a retracted article, indicate the article's retracted status in the References list and also include a citation and full reference for the retraction notice.

Reply: We reviewed our reference list and corrected it if needed. Furthermore, we added reference number 22. This paper was not published when we submitted the article, but is now published.

We made two other general changes as well.

- *The notation of the **affiliation of Hasselt University** was incorrect. We changed Faculty of Rehabilitation Sciences and Physiotherapy into Faculty of Rehabilitation Sciences.*
- *We added the correct **data availability statement**, as was emailed to you on October 6th 2023.*

  This now reads:

  Following documents are open access available in the UK DataService ReShare repository (10.5255/UKDA-SN-856735): 1. the codebook and memo generated during analyses, 2. the full interview guides of the individual and focus-group interviews, and 3. the informed consent forms. The interview transcripts generated and analysed during this study are not publicly available due to their containing information that could compromise research participants' privacy/consent.

Reviewer comments:

Reviewer #2

I am happy with how you have handled my concerns and I hope your manuscript will be accepted for publication.

Good luck, Mats

Thank you for the compliments.

Reviewer #3

Thank you for your hard work to revise this manuscript. I believe it is well written and well organized with valuable information. It is a much improved manuscript. I have very few comments below.

Thank you for the compliments and your feedback.

Table 3

1)Therapists' teaching style: PPT characteristics, is there a typo, "Now, IT THINK IT MOSTLY because it suits me."

2)Motivation: Motivation as a prerequisite for learning is there a typo, "If they fail TO often….."

Reply: We corrected the typos

This now reads:

- *… I think it is mostly …*
- *… fail too often …*

Good comments in the Discussion about the OPTIMAL theory as it related to your data. It does not appear that PPT's reported on one of the third areas of content in the OPTIMAL theory, using an external vs. internal focus of attention. I'm assuming that is why this area was not considered in the Discussion.

Reply: You have interpreted it correctly. The PPTs agreed that attention for motivation was highly relevant in children with DCD, as supported by the OPTIMAL theory. The OPTIMAL theory also suggests to adopt an external focus of attention. However, as mentioned in section r500-509 therapists used both, and literature in children's motor learning has not yet been able to demonstrate that an external focus of attention benefits children's motor learning more than an internal focus of attention (e.g. van Cappellen-van Maldegem et al. 2018, Simpson et al. 2020, van Abswoude et al. 2021).

Would it be appropriate to add "Enhanced Expectancies" to the Instructions and Feedback section of the SI document as it is one of the pillars of the OPTIMAL theory that is not reflected. Given this theory, I might add to Guided Discovery in S1, by reflecting it as "Autonomy/Guided Discovery."

Thank you for this suggestion, we added "Enhanced Expectancies" to the S1 file. Furthermore, we added "self-controlled" in the sections instruction and feedback, and organization of practice, for the autonomy part of the OPTIMAL theory. As a consequence, an extra reference was added to the reference list, changing the numbers of the references.

This now reads in the Instructions and feedback section:

**Instructions and feedback**	
Self-controlled	In self-controlled instructions and feedback the learner determines the timing or modality of the instructions and feedback, which enhances the learner's autonomy [2].

Enhanced Expectancies	Instructions and feedback that enhance the learner's self-efficacy expectations (or confidence) about the movement performance [2].

In the organization of practice section, this now reads:

Self-controlled	In self-controlled practice conditions the learner is provided with choice, which enhances the learner's autonomy [2].
